# Breaking the mid-infrared interconnection barrier: a robust bonding for high-power optics based on liquid-like chalcogenide glass

**DOI:** 10.1038/s41377-025-02098-0

**Published:** 2026-03-02

**Authors:** Xiange Wang, Feng Xiao, Yiming Du, Kai Jiao, Keke Chen, Wei Tang, Yuyang Wang, Xiang Shen, Shixun Dai, Maozhi Li, Xunsi Wang, Shengchuang Bai, Rongping Wang, Ganapathy Senthil Murugan, Barry Luther-Davies

**Affiliations:** 1 Laboratory of Infrared Material and Devices, The Research Institute of Advanced Technologies, College of Information Science and Engineering, Ningbo University, Ningbo, 315211 China; 2 College of Information and Intelligence Engineering, Zhejiang Wanli University, Ningbo, 315000 China; 3 Department of Physics, Beijing Key Laboratory of Opto-Electronic Functional Materials & Micro-Nano Devices, Key Laboratory of Quantum State Construction and Manipulation (Ministry of Education), Renmin University of China, Beijing, 100872 China; 4 Optoelectronics Research Centre, University of Southampton, Southampton, SO17 1BJ United Kingdom; 5 Deparment of Quantum Science and Technology, The Australia National University, Canberra, ACT 0200 Australia

**Keywords:** Mid-infrared photonics, Integrated optics

## Abstract

Achieving low-loss optical interfaces between high-refractive-index (*n* > 2) components is critical for mid-infrared photonic systems, yet hindered by the trade-off between refractive index matching, IR transparency and thermal stability. Here, we introduce a groundbreaking solution—bonding the optical lenses and fibers with a liquid-like chalcogenide glass, which possesses an ultra-low glass transition temperature below room temperature, high refractive index and exceptional mid-infrared transparency. The basic performances of the liquid glass are characterized and proved by detailed viscosity distribution, mechanical shear and bonding tensile strength measurements. Most of all, the optical transmission and laser delivery of these bonded chalcogenide glass fiber devices demonstrate a significant improvement, with transmission efficiency increasing from 36% to 91%, and laser power delivery from several hundred mW rising to 14.5 W at a wavelength near 4 µm. Additionally, the system demonstrates long-term stability, maintaining performance over at least 3 months and more than 206 heating-cooling cycles when utilizing this liquid-like glass adhesive. This research not only addresses the challenge of bonding mid-infrared optical components but also holds immense promise for advancing integrated mid-infrared optics applications, including spectroscopy, sensing, and imaging.

## Abstract (web summary)

Liquid-like chalcogenide adhesive enables robust mid-IR fiber bonding, eliminating refractive index mismatch and organic absorption, achieving efficient 11.7 W laser power delivery at 4.7 µm with record performance.

## Graphical abstract

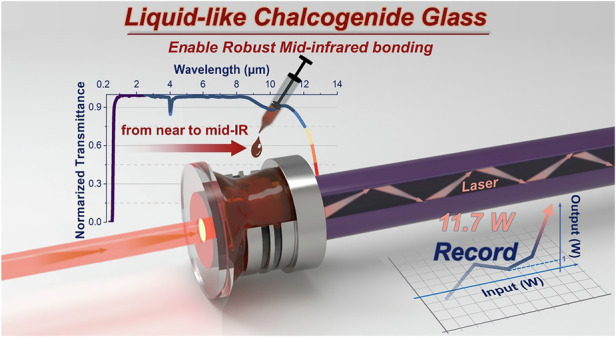

## Introduction

The mid-infrared (MIR) spectral region has emerged as a critical technological frontier, enabling groundbreaking applications in sensing, medicine, and industry^1,2^. These advanced applications demand optical systems capable of high-power transmission, compact integration, and long-term stability. However, the performance of MIR photonic systems is fundamentally constrained by two persistent challenges: (i) substantial Fresnel losses at high-refractive-index (*n* > 2) optical interfaces (e.g., Ge, ZnSe, chalcogenide glasses)^3,4^, and (ii) the absence of reliable, low-loss interconnection technologies for compact system integration.

Current approaches to minimize interfacial reflections primarily rely on anti-reflection (AR) coatings^5^ or biomimetic moth-eye micro-structures^6^. While AR coatings are theoretically effective, they are prone to damage under high-power laser irradiation due to a mismatch in the coefficient of thermal expansion (CTE) between solid materials^7^. Biomimetic moth-eye structures, despite their broadband anti-reflection potential, lack experimental validation for high-power MIR laser applications owing to their inherent structural fragility^6,8^. More critically, these solutions only address single-surface reflections while failing to resolve the fundamental challenge of achieving low-loss, compact optical interconnects between discrete components.

In visible and near-infrared systems, optical adhesives (e.g., epoxies, UV-curable polymers) are widely employed for optical components attachment^9–16^. However, their organic or related molecular structures containing necessary active hydroxyl, carbonyl, oxide groups or other impurities^17,18^ exhibit strong intrinsic absorption across key MIR atmospheric windows. Furthermore, the severe refractive index mismatch (e.g., *n* ≈ 1.5 for NOA61 optical adhesive produced by Thorlabs Co. vs. *n* ≈ 4 for Ge lens) generates interface reflection losses exceeding ~19%, rendering them unsuitable for high-power MIR applications. The attempts in the development of high-index alternatives for applications in the infrared, such as sulfur-containing polymer systems or sol-gel-derived inorganic gels^19–24^, have their own limits. For instance, sulfur-containing polymers, while providing refractive indices of ~1.6–1.8 in the visible/near-infrared, suffer from C-H/O-H absorption beyond 2.5 μm, insufficient index matching for high-index MIR optics, and low tolerance to high-power irradiation due to limited polymer matrix stability. Similarly, sol-gel materials, while transparent in certain infrared bands, generally lack broadband mid-infrared transmission. They are often brittle and prone to cracking due to volume shrinkage during the curing process.

Fusion splicing technology faces its own inherent limitations. The significant melting temperature difference between common MIR materials (e.g., Crystalline Ge vs. chalcogenide glasses) leads to thermal stress fractures, while the technique’s poor geometric adaptability hinders reliable bonding of complex heterostructures (e.g., lens-fiber-chip assemblies)^25–27^. These fundamental limitations highlight the urgent need for a transformative interconnection technology that achieves low optical loss, high-power tolerance, and thermal stability in compact MIR systems.

Chalcogenide glasses have garnered significant attention due to their exceptional MIR transparency (beyond 12 μm), tunable refractive index (*n* = 1.8–3.5), and low glass transition temperatures (*T*_*g*_ usually below 300 °C)^28,29^. Since Flaschen’s pioneering report on As-S-I glass (*T*_g_ ≈ 50 °C) in 1960^30^, the chemical stability of chalcogenide glasses has been enhanced through Se doping^31^, and their preliminary applications have been demonstrated in diamond defect detection^32^ and LED radiation enhancement for refractive index matching^33^. However, to date, there have been no reports on the laser irradiation and structural stability of these bonded optical glass devices, particularly for high-power optical lens or fiber-integrated systems in critical MIR applications, which require easier to adhere (lower *T*_g_), reliable heterojunction interface connections, mechanical stability, and high-power laser handling capabilities.

In this study, we introduce a breakthrough liquid-like chalcogenide glass bonding technology using an optimized As-S(Se)-I system with precisely controlled glass transition temperature and refractive index. The engineered liquid-like material exhibits superior adhesive and laser anti-irradiation properties, without the risk of cracking under thermal shock from lasers. It also enables complete interface conformal filling and high delivery efficiency of up to 91%, even at high temperatures (120 °C) and under high laser powers (14.5 W), transitioning into a robust inorganic glass state upon heating and cooling, with exceptional bond strength. This innovative approach facilitates high-efficiency coupling of infrared lens-fiber heterogeneous assemblies, delivering a mid-infrared high-power laser output of 11.7 W. The integrated systems demonstrate remarkable reliability in long-term thermal shock tests, undergoing 206 cycles of thermal cycling from 25 °C to 120 °C over a period of 3 months. This technological advancement establishes a universal integration platform for next-generation mid-infrared photonic systems, providing a transformative solution for high-density component integration and high-power laser delivery applications.

## Results

### Liquid-like glass optimization, preparation and characterization

**Fig. 1:**
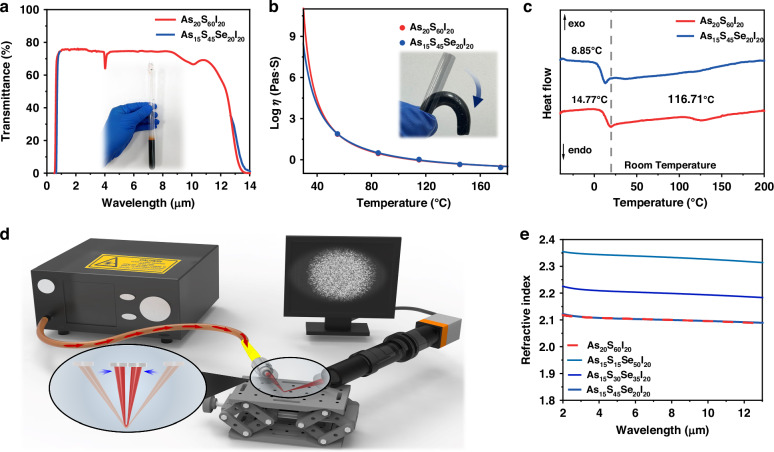
Characteristics of the liquid-like glass. **a** Transmission spectrum of the chalcogenide glass As_15_S_45_Se_20_I_20_ and As_20_S_60_I_20_ with a thickness of ~2 mm, the inset is the glass sample. **b** The measured viscosities for glass As_15_S_45_Se_20_I_20_ and As_20_S_60_I_20_ as a function of temperature: the blue and red lines are the fitting based on Vogel–Fulcher–Tammann (VFT) method, the inset is the bendable glass rod. **c** DSC of the liquid-like glasses of As_20_S_60_I_20_, As_15_S_45_Se_20_I_20_ glasses. **d** The setup to examine the light transmission ability of the “V” shaped fiber. The output beam profile is evident on the monitor. **e** Refractive indices of the As_20_S_60_I_20_, As_15_S_15_Se_50_I_20_, As_15_S_30_Se_35_I_20_, As_15_S_45_Se_20_I_20_ glasses

A series of liquid-like mid-infrared As_x_S_y_I_z_ (10 ≤ x ≤ 50, 30 ≤ y ≤ 90, 0 ≤ z ≤ 30, S can be partly or all replaced by Se^34^) glasses were prepared by the traditional melt-quenching method, as their glass-transition temperatures are all below the room temperature. The detailed glass composition, *T*_g_ and the refractive index of As_15_S_(65-x)_Se_x_I_20_ can be found in Supplement 1, Supplementary Table S1. During the glass preparation, impurities related to C-, H-, and O- can be effectively removed from the glass through a purification process (Supplement 1, S1). As two typical liquid-like glasses, As_20_S_60_I_20_ and As_15_S_45_Se_20_I_20_, they can tightly adhere to the wall of the quartz tube under the room temperature. According to the transmission spectrum shown in Fig. 1a and the extinction coefficient data (Supplement 1, S2), the glass demonstrates excellent optical transparency across the 0.7–10 μm wavelength range. A minor absorption feature near 4.1 μm is attributed to H-S bonds commonly found in chalcogenide glasses. There are only slight red-shifts of Se-contained glass both in the short and long cut-off wavelength, so the Se-contained glass looks like a darker red transparent liquid (inset of Fig. 1a). These two kinds of glasses show nearly the same viscosity-temperature curve (Fig. 1b) with a steep distribution of viscosity varying, i.e. a typical value of 10^8.3^ Pa·s at room temperature of 30 °C and this gradually decreases to 10^−0.08^ Pa·s at 120 °C which are comparable to the common organic optical adhesives used in bonding optical elements. The inset shows that the Se-contained glass can be freely bent by hand at 55 °C, with a viscosity of 10^1.9^ Pa·s.

To investigate the internal structural changes of the glasses, the Raman scattering spectra of As_40_S_60_, As_20_S_60_I_20_ and As_15_S_45_Se_20_I_20_ glasses were compared, as illustrated in Supplement 1, S3. Raman scattering spectra together with the assignment of each peak indicated that these glasses mainly contained S-S and As-I bonding, but the existence of As-S and S_8_ ring structures is also evident^35–37^. A striking feature is that the ratios of Raman intensities of S_8_ and As-I peaks to that of As-S peak become large in the liquid-like glasses. It is well known that the halogen element ‘I’ can cut the chemical bonds due to its relatively strong ionic feature^38^, and thus break the network, leading to the soft glasses of As_20_S_60_I_20_ and As_15_S_45_Se_20_I_20_. The detailed softening process and glass structure can be simulated, and the case of As_20_S_60_I_20_ clearly shows that most As and S atoms have 3 and 2 nearest-neighbor atoms, respectively (insert in Supplement 1, S4a). According to the partial pair distribution functions, the coordination number of almost all I atoms is only 1, as shown in Supplement 1, S4b. The structure of the glass indicates that introducing I atoms break the connection of the glass network, leading to the liquid-like behavior of As_20_S_60_I_20_ glass. The same applies to As_15_S_45_Se_20_I_20_.

The primary effect of this structural change is the alteration in the glass transition temperature. As shown in Fig. 1c, As_20_S_60_I_20_ exhibits two glass transition temperatures, 14.77 °C and 116.7 °C, within the range of −40 to 200 °C. This indicates that the glass of this composition undergoes significant phase separation, and prone to hydrolysis or depolymerization, which is the main reason for its instability and limited usability. However, its relatively high transparency in visible spectrum can increase the observability of experiments with human eyes, thus, this kind of glass can be employed as an optical adhesive for short-term optical experiments.

In contrast, the addition of Se to the glass effectively inhibits crystallization and phase separation, and the As_15_S_45_Se_20_I_20_ glass has only a single, low glass transition temperature of 8.85 °C. Not only this lower transition temperature creates favorable conditions for glass bonding, but also the glass can maintain good transparency even after long-term storage of 1 year under room temperature and atmosphere conditions (Supplement 1, S5). Then, the loss of the weight was measured after the glass was placed in a high-temperature environment of 120 °C for 5 min, without protecting gas condition, as shown in Supplement 1, S6. After total 37 heating cycles, there is only 0.1% weight loss under a continuous high temperature of 120 °C and total time up to 185 min, except for some volatilization at surface. This proved that the As_15_S_45_Se_20_I_20_ glass is stable enough for next operation of heating bonding and cold adhering even at an open environment. These results can lay a solid foundation for the material being utilized in complex environments, including repeated high-temperature and high-power laser bonding experiments.

Moreover, the liquid-like properties of the glass provide it with excellent ductility. When the temperature exceeds 55 °C, the glass can be easily drawn manually without experiencing stress fractures. The glass could also be folded and bent sharply as shown by the “V”-shape (Fig. 1d right-down). The output beam profile from this structure clearly demonstrates that this sharply folded fiber can guide light with a flexibility not achievable using conventional fibers (Fig. 1d right-up). The light guiding is supported by the consistently high refractive index (RI) of greater than 2 across the entire wavelength range from 2 to 12 µm. The RIs of As_20_S_60_I_20_ and As_15_S_45_Se_20_I_20_ are nearly the same (2.1 at a wavelength of 4.7 µm), as shown in Fig. 1e. All these above characteristics hold potential for application in flexible wearable devices and miniaturized sensors.

### Adhesive performances based on liquid-like glass

**Fig. 2:**
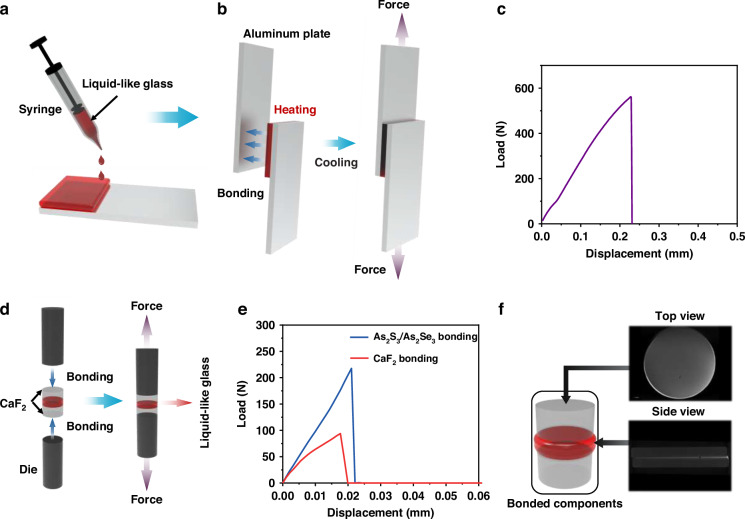
Adhesive performance of the liquid-like glass. **a** Spreading liquid-glass. **b** Left view of bonding aluminum plates using the liquid-glass, right view of shear measurement of the load as a function of the load cell displacement. **c** Relationship between displacement and shear load. **d** Left view of diagram of lens bonding, right view of tensile strength of the bonded lens group. **e** Relationship between displacement and tensile load. **f** Two CaF_2_ glued using the liquid-glass, top view of the facet of the bonding group by computed tomography scanning, side view of the facet of the bonding group by computed tomography scanning

To measure the mechanical properties, the liquid glass in the syringe was heated to 120 °C and evenly applied to the surface of the bonding material as shown in Fig. 2a, where another object was then easily attached under pressure. Figure 2b is an example where two aluminum plates with a contact surface of 25 × 25 mm were successfully bonded for the measurement of the shear force. The optical adhesive was used to successfully bond. When the bonded materials were glass slides, they would be broken before separation during the measurement. Therefore, the aluminum plates were used for the measurements. The test was performed according to ASTM D3039 standard. When a load force of 561.8 N was applied, the aluminum plates were separated, resulting in failure at the bonded interface (Fig. 2c).

Figure 2d is a schematic diagram of the bonding tensile strength measurement. Separation occurred when the load forces of 93.9 N and 217.6 N were applied for the bonded CaF_2_ lenses, as well as the bonded As_2_S_3_ and As_2_Se_3_, respectively, as shown in Fig. 2e. The tensile strength reached 28.25 kg/cm^2^ and kept at the same level even after 7 days following bonding, which is comparable with the data of a traditional UV-Curable Noa 61 adhesive after 7 days storage^39^.

To evaluate the reliability of adhesive performance, the CaF_2_ bonded components were tested in accordance with the MIL-A-3920C standard. As shown in Fig. 2f, a small number of bubbles formed within the bonded area after testing, with the coverage of the bubble area being less than 1% (Supplement 1, S7). The bond remained intact, exhibiting no edge feathering, separation, or voids. All these characteristics highlight its capability to bond various optical elements, including lenses, waveguides, optical fibers, and thus the liquid-like glasses were investigated as an optical adhesive in the subsequent section of this paper.

### Interface reflection reduction via Liquid-like glass gluing

**Fig. 3:**
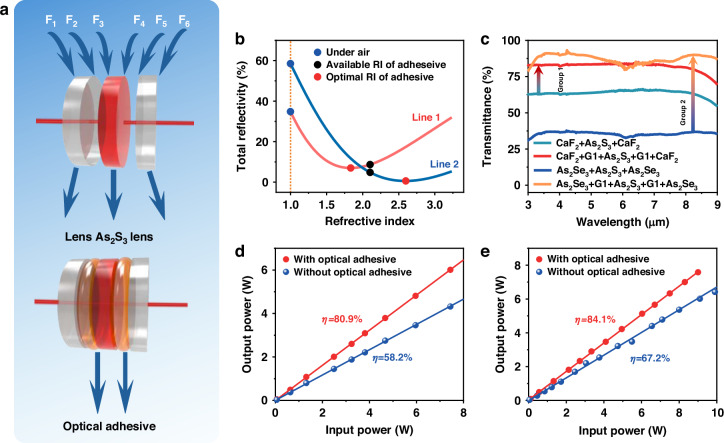
Infrared lenses bonding. **a** Sketch of all six faces on lenses before and after bonding. **b** Fresnel reflectivity vs. RI of the filling media. **c** Experimental FTIR transmission in lenses with and without the optical adhesive. **d** Laser delivery of the group of the lenses at 2.94 µm. **e** at 4.7 µm, with and without the optical adhesive

To evaluate the bonding potential of this liquid-like glass, we conducted a transmission study comparing the effect of two groups of bonding with and without optical adhesive on the As_2_S_3_ glass transmittance, as shown in Fig. 3a. The first group (Group 1) consists of an As_2_S_3_ glass lens (2 mm thickness, 10 mm diameter) and two CaF_2_ crystal lenses (1 mm thickness, 10 mm diameter), with a 0.1 mm optical adhesive layer. The second group (Group 2) includes an As_2_S_3_ glass lens (2 mm thickness, 10 mm diameter) and two As_2_Se_3_ planar lenses (each coated with a single-side anti-reflection film, 2 mm thickness, 10 mm diameter, see Supplement 1, S8), also with a 0.1 mm optical adhesive layer. The air interfaces (F1–F6) cause significant reflections, particularly from the As_2_S_3_ surface due to its high refractive index (RI). Therefore, filling the gap between the lenses with a high RI optical adhesive helps reduce the total reflection loss. Using the Fresnel formula, we calculated the total reflection for the lens groups with four interfaces (F2, F3, F4, and F5) as a function of the RI of the filling medium (Fig. 3b). In line 1, corresponding to lens Group 1, the largest reflectivity occurs when the gap is filled with air. As the RI of the filling medium increases from 1 to 1.83, the reflectivity decreases from 34.8% to 7.0%, then gradually increases. A filling medium with an RI of 1.83 minimizes reflection, and the liquid-like glass used in this study, with an RI of 2.1, results in a reflectivity of only 8.7% (black dot), which is close to the optimal scenario, representing a reduction in reflectivity by a factor of ~4. In line 2, corresponding to lens Group 2, the largest reflectivity also occurs when the gap is filled with air. This reflectivity decreases from 58.5% to 0.59% as the RI of adhesive increases from 1 to 2.58, and then slightly increases. A filling medium with an RI of 2.58 is optimal, and the liquid-like glass with an RI of 2.1 results in a reflectivity of only 4.8% (black dot), a reduction by a factor of ~12.2.

Experimental transmission results for the bonded lens groups, with and without the optical adhesive, are shown in Fig. 3c. In the 3–9 μm wavelength range, the transmission of group 1 (CaF_2_ + As_2_S_3_ + CaF_2_) is only ~62%, which can be derived from the ~90% transmission for CaF_2_ lens and ~68% for the As_2_S_3_ lens in air. When the interfaces F2, F3, F4, and F5 are filled with the liquid-like chalcogenide glass adhesive, transmission increases to ~83%, although improvement is limited by the Fresnel reflections on the outer surfaces F1 and F6 of CaF_2_.

For Group 2, the AR-coated As_2_Se_3_ glass exhibits a higher concentration of H_2_O-related impurities than As_2_S_3_ glass—largely due to differences in processing and purification. These residual water molecules generate a vibrational absorption band near 6.3 µm^40^, causing a modest dip in transmittance. Despite this, the bonded glass’s overall transmittance improves dramatically—from roughly 36% to 91%—approaching the theoretical limit for low reflection (~95.2% transmittance, corresponding to 4.8% reflection). The remaining ~4% loss is attributable to residual reflections from the coated surfaces of F1 and F6. This dramatic enhancement demonstrates the effectiveness of our bonding technology in reducing interfacial losses while maintaining excellent optical transmission characteristics. Although some discrepancies remain between the experimental results in Fig. 3c and the theoretical evaluation in Fig. 3b, due to factors such as impurity absorption, surface scattering, and measurement errors, these are the first results showing the effective use of liquid-like glass as a reflection-reducing medium for bonding materials with a large RI contrast, thereby enhancing the transmission of the bonded optical components (in this case, the lens group). Besides, this bonding method is applicable to a broader range of infrared optoelectronic materials, such as Ge, which typically has a higher refractive index (~4). Theoretical verification of transmittance improvement for Ge is presented in Supplement 1, S9a. Assuming negligible material absorption, the transmittance of Ge can be increased by over 70% above 1 μm, reaching a maximum of ~71%. As shown in Supplement 1, S9b, experimental validation further supports this result: the measured transmittance after bonding (69% at 4.7 μm) shows a 45% enhancement compared to that before bonding (47.6% at 4.7 μm).

In addition to its optical performance, the proposed bonding glass demonstrates excellent thermal and chemical stability. After 10 heating cycles at 120 °C, no noticeable increase in As-O absorption was observed (see Supplement 1, S10a), confirming negligible oxidation. Although the glass itself displays moderate hygroscopicity under high-humidity conditions (see Supplement 1, S10b), the encapsulation provided by bonding effectively mitigates moisture-induced degradation, ensuring excellent water resistance in practical applications (Supplement 1, S10c). Combined with its strong anti-reflective properties and material compatibility, these features highlight the broad applicability and long-term reliability of the proposed bonding approach for advanced mid-infrared photonic systems.

To verify whether the optical adhesive improves the efficiency of power delivery under high-power conditions, we further examined the power delivery of the lens groups before and after bonding at 2.94 µm and 4.7 µm with collimated light, as shown in Fig. 3d, e. The delivery efficiency of the bonded optical component was 80.9% at 2.94 µm and 84.1% at 4.7 µm, representing an increase of ~22.7% and ~16.9%, respectively, compared to the free-space lens group. The slight difference between the transmission and power delivery results is due to the higher accuracy of the power delivery measurements. Nevertheless, all results confirm that liquid-like glass plays a crucial role as an optical adhesive, effectively reducing reflection losses and improving transmission in high-power laser systems.

### Laser power delivery via Liquid-like glass gluing

This clearly demonstrates that the liquid-like glass can be used as optical adhesive in the mid-infrared. We also verified the effectiveness of bonding between single-mode silica fiber and an As_2_S_3_ multi-mode fiber fitted with FC connectors using the liquid-like glass (As_20_S_60_I_20_), where we used the coupling from single- to multi-mode fiber in order to reduce the effect of the alignment on the coupling efficiency (Supplement 1, S11). Coupling back from the multi-mode As_2_S_3_ fiber to the single-mode silica fiber was not considered in the paper. The delivery efficiency between the fibers was improved by 17.3%, which is close to the estimate based on the reduction in Fresnel losses.

Enhancing the delivery efficiency through bonding fibers or lenses with this liquid-like glass raises another pivotal question: can the use of this glass improve the power handling capacity of an optical fiber from a mid-infrared laser? This is especially an issue for chalcogenide glass fibers, given that their weak covalent bonds usually result in particularly low surface damage thresholds, typically at the level of kW/cm^2^. In order to mitigate this problem when coupling high laser power to chalcogenide fibers, we designed a fiber taper that reduced the incident power density at the chalcogenide surface and was bonded, using our liquid-like glass, to a crystalline window (CaF_2_) which has a high laser damage threshold and low RI as a fiber endcap to replace the air interface just the same as Fig. 3, and we examined the laser delivery in such a system.

We simulated the delivery efficiency of the assembly with different tapers, and found that the best taper had an outer diameter of 4 mm and a fiber core diameter of 2 mm at the input and a “tail” fiber core diameter of 500 μm at the output and a taper length of 4 cm (Supplement 1, S12a). In this case, the simulated delivery efficiency was about 94% (Disregarding Fresnel reflection). The NA and minimum loss of the endcap were measured to be 0.34 and 0.1 dB m^−1^ at 4.7 μm, respectively (Supplement 1, S12b).

**Fig. 4:**
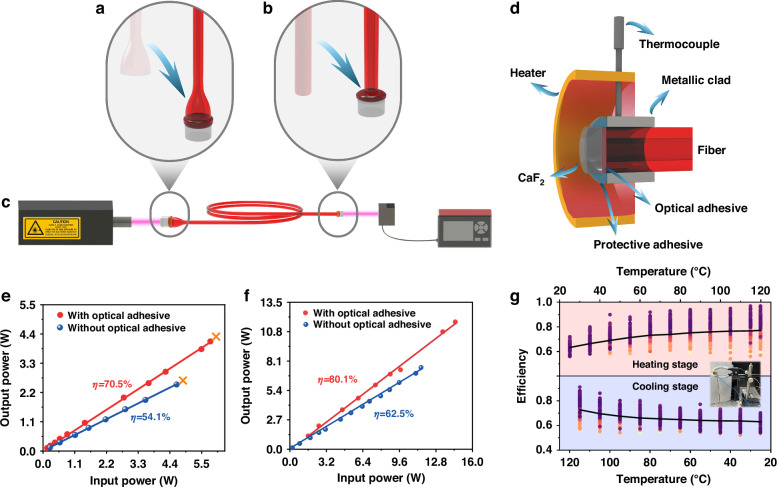
Mid-infrared fiber endcap bonding. **a** Sketch for fiber endcap bonding with lens. **b** Bonding the output fiber facet with lens. **c** Setup of the fiber laser power delivery. **d** Schematic diagram of the end cap package. **e** Laser delivery at 2.94 µm. **f** Laser delivery at 4.7 µm with and without bonding. **g** Transmission vs. working temperature at an input power of 100 mW at 4.7 μm. A total of 206 measurements were conducted to evaluate thermal stability, with each data point shown as a colored circle

As shown in Fig. 4a, b, the main process of bonding the fiber taper to the endcap is illustrated, and the laser delivery was measured. First, the liquid-like glass in a syringe was preheated to ~120 °C. Then, a drop of the glass was carefully squeezed onto the top of a CaF_2_ lens preheated to 80 °C, forming an ellipsoidal layer, as shown in Fig. 2a. The purpose of the preheating was to reduce the glass viscosity for easier coverage of all the surface and improve the adhesion. Then the heater for the CaF_2_ was switched off and the fiber taper was quickly pressed onto the CaF_2_ cap (Fig. 4a), forming an adhesive layer ~0.1 mm thick. The bonding process was optimized by fine‑tuning both the direction and magnitude of applied forces during assembly, ensuring uniform layer thickness and effectively suppressing bubble formation to achieve excellent optical contact. A well‑formed, consistent bonding interface also enables precise mechanical packaging after curing—critical for maintaining structural integrity and preventing deformation or misalignment under thermal stress. This optimized process guarantees both superior optical performance and long‑term mechanical robustness. The same processing procedure was applied to the bonding between the output facet of the fiber tail and CaF_2_ (Fig. 4b). Finally, a setup to measure the delivery efficiency and power was built (Fig. 4c), where the laser was incident onto the fiber endcap and then collected by the power meter via the output tail end of the fiber.

To ensure that the bonded fiber endcap can withstand thermal shock and enhance stability, the overview of the fiber endcap in a metal enclosure, as illustrated in Fig. 4d, was designed. The fiber endcap was inserted into a metal tube, with the connection sealed and secured using protective adhesive (K704, Kafuter). This well-engineered enclosure offered robust support for controlling the shape of the adhesive and maintaining system stability. The As_2_S_3_ fiber endcap was enclosed by a wrap-around heater, and a thermocouple was positioned in proximity to the metal tube surrounding the fiber endcap. Stable transmission efficiency was recorded by adjusting the heater temperature while monitoring power output using a power meter.

The power delivery efficiency of the fiber taper with and without the optical adhesive were measured (Fig. 4e). A 2.94 μm mid-infrared laser with a beam diameter of 1.0 mm, a repetition rate of 15 Hz and a pulse width of 400 μs was coupled into the As-S fiber taper. In the case without optical adhesive, the delivery efficiency was 54.1% with a maximum output average power of 2.5 W, while in the case with the optical adhesive, the delivery efficiency increased to 70.5% with a maximum average output power of 4.2 W. The input fiber taper did not show any damage due to the low surface power density, however, the tail end of the fiber showed thermal damage due to an absorption of OH^-^ at ~2.9 μm as well as poor heat dissipation at the tail end.

The laser delivery efficiency and power at 4.7 μm with and without the optical adhesive were measured (Fig. 4f). The beam from 3.7 to 4.7 μm mid-infrared solid-state laser (WP-LA-Ⅳ05/M20) with a repetition rate of 10 kHz, a pulse width of 60 ns, a spot diameter of 1 mm, and a maximum average power of 14.5 W was coupled into the input fiber. Without the optical adhesive, the delivery efficiency was ~62.5% with a maximum output of 7.51 W, where the maximum working temperature in the conical region of the endcap was 145 °C. But this increased to 80.1% stably with a maximum output of 11.7 W in the case of the system with the optical adhesive. The distribution of the temperature was recorded by an Infrared Thermal Imager (T3, DALI Technology Co., China) (Supplement 1, S13), where the maximum temperature in the conical region of the endcap was 113 °C when working at the maximum input power. The taper contributes to most of the loss and the light lost in this region was absorbed by a PES polymer on the surface of the fiber taper and this led to the high temperature in the conical region. The method of preparing the taper made it impractical to remove the PES coating for these experiments but clearly this should allow the surface temperature to be reduced in future designs. Nevertheless, such a temperature is still less than *T*_*g*_ of As_2_S_3_ around 180 °C, indicating that there still is room to further increase in the input laser power that was mainly limited by the laser available to us for these experiments. The delivery efficiency and maximum output power in the multimode fiber are 80.1% and 11.7 W, respectively. Compared with no optical adhesive, the transmission efficiency increased by ~28%. Moreover, the bonded fiber device enables a 167-fold enhancement in delivery power compared to traditional mid-infrared coating film^7^, thanks to the effective stress-relieving liquid-like adhesive at the interfaces.

In order to verify the effect of the working temperature on the transmission of the bonded fiber endcap, we examined the stability of power delivery in a 20-cm-long As-S fiber endcap at different temperatures at 4.7 μm. The main panel (Fig. 4g) presents the temperature-dependent transmission efficiency measured over 206 heating-cooling cycles conducted over three months. The upper half of the figure, highlighted in red, represents the heating process, while the lower half, shaded in blue, denotes the cooling process. The black curve shows the average transmission efficiency across all 206 cycles. Due to the proximity of the end face of the fiber to the thermistor, the efficiency variation during the heating and cooling phases is also influenced by the ambient temperature increase caused by the heater. It can be observed that the efficiency showed little change over different cycles, confirming the thermal stability of the assembly.

To understand the origin of improved power delivery when using the optical adhesive (As_20_S_60_I_20_), we conducted a separate measurement using the 2.94 and 4.7 μm lasers to determine the damage behavior of As_2_S_3_ bulk glasses and tapers bonded with and without CaF_2_ lens on the input facet. No damage was observed under 4.7 μm irradiation. When exposed to a 2.94 μm laser with low repetition rate and high pulsed energy, typical damages were observed in the glass and fiber taper. The damage, as well as the maximum input and output pulsed energy density in the glasses and fiber tapers were presented, respectively, at 2.94 μm (Supplement 1, Supplementary Table S2). The damage occurs at the input facet in the case of the glass without optical adhesive (Supplement 1, S13a), and this changes to output facet with the optical adhesive (Supplement 1, S13b). Both the maximum input and output pulsed energy densities increase when using the optical adhesive by more than 59%. In the case of fiber taper, the maximum output pulsed energy density (compared with their respective maximum energy input) increases due to the small beam size of the output light. The damage occurs at the taper region because of the leakage of the light into the polymer coating, where strong absorption leads to the damage (Supplement 1, S13c). The output pulsed energy density increases by about 65% with the use of optical adhesive. Overall, the data indicates that the optical adhesive contributes to the increased damage threshold, leading to high-power delivery. The similar results have been reported in ref. ^41^ for 1.06 μm laser delivery.

## Discussion

In summary, we have developed a novel chalcogenide glass bonding technology based on an As-S(Se)-I system with an ultralow glass transition temperature (*T*_*g*_ = 8.85 °C) and a tailored refractive index (*n* ~ 2.1). The engineered material exhibits unique behavior at a modest temperature of 120 °C, enabling effectively fills interfacial gaps, while forming a robust glass interconnection upon cooling with excellent mechanical strength. Using this adhesive, transmission is improved from ~36% to ~91% in As_2_S_3_ + As₂Se₃ bonded lenses (G1), and from 62% to 83% in As_2_S_3_ + CaF_2_ bonded groups (G2), achieving a minimal reflection of only 4.8%, down from 58.5% in traditional free-space coupling optical systems. Additionally, the laser damage threshold increases by more than 59%. Most critically, the laser delivery power of an endcap fiber bonded with CaF_2_ lenses reaches 11.7 W, marking a 167-fold enhancement compared to traditional mid-infrared film-coated fibers. This approach enables high-efficiency coupling of heterogeneous lens-fiber components, delivering high laser output and exceptional thermal stability. It represents the first demonstration of low-*T*_*g*_ chalcogenide glass as a high-performance optical adhesive for mid-infrared systems, overcoming the limitations of conventional organic adhesives and high-temperature fusion techniques in this domain. Furthermore, the unique combination of thermoplastic processability and stability of the liquid-like glass offers unprecedented flexibility in photonic packaging, while maintaining a high laser damage threshold. This technology establishes a universal platform for the heterogeneous integration of high-index mid-infrared components, a critical capability for advancing applications in chemical sensing and infrared countermeasures. With its exceptional performance, this technology opens new pathways for compact, high-power mid-infrared photonic systems.

## Materials and methods

### Glass preparation and purification

The glass preparation and purification processes can be described as shown in Supplement 1, S1. High purity 5 N (99.999%) As and S materials were accurately weighed, and put into a silicon dioxide ampoule labeled as A1 together with a deoxidizing agent of Mg. The ampoule was subsequently evacuated to 10^−5 ^Pa. This assembly was subjected to heating to eliminate any water molecules adhering to the surface of the raw materials. Following the sealing of the ampoule, it underwent a comprehensive reaction within a rolling furnace to eliminate any O-related impurities from the raw materials. Once this initial phase was complete, the A1 ampoule was connected to A2–A5. Initially, A1 was heated to enable the raw materials to be distilled into A2 due to the pressure difference, while the solid impurities like MgO and metallic Mg were retained in A1. After distillation, the connection between A1 and A2 was sealed. Subsequently, A2 and A3 were heated to distill As, S, and I source materials into A4 for further purification, and the connection was subsequently sealed. Finally, the raw materials housed in A4 were distilled into A5 to eliminate nearly all impurities. The purified materials were then mixed and melted within a rolling furnace at 650 °C for a duration of 10 h, taken out at 400 °C, following by water quenching, subsequently annealing at a furnace maintained at around 100 °C, and finally cooling down to room temperature.

### Transition temperature of glasses

The glass transition temperatures (*T*_*g*_) of G1-G4 glasses were measured using a differential scanning calorimetry (NETZSCH DSC 214) at a heating rate of 10 °C/min over a temperature range from −50 °C to 200 °C.

### Oxidation test

Oxidation tests were conducted on As-S(Se)-I glass samples with a diameter of 13 mm and a thickness of 3 mm. Each sample was bonded to a CaF_2_ window on one side, with the opposite side left exposed for direct heating. The samples underwent ten heating cycles at 120 °C, each lasting ~60 s. To assess potential oxidation, relative spectral analysis was performed by comparing the absorption intensities of the characteristic As-O peaks (15.4, 12.7, and 9.5 μm) to the strong S-H absorption band at 4 μm, as shown in Supplement 1, S10a.

### Water-resistant tests

Two sample configurations were immersed in water for five days: (1) As-S(Se)-I glass bonded to a 2 mm KBr window on one side, and (2) the same glass fully encapsulated between two 2 mm CaF_2_ windows. In the partially exposed sample, surface water could not be wiped away but was removed by gentle heating. Transmittance changes were monitored daily for both configurations.

### Fabrication of the fiber taper

High-purity As-S core glass (As_40_S_60_) and cladding glass (As_38_S_62_) with a diameter of 23 mm and 46 mm, respectively, were used to fabricate the preform utilizing the isolated peeling-off extrusion technique as described in ref. ^42^. Subsequently, the preforms, after being cut into 5 cm length, underwent a process of being coated with two layers of polyethersulfone (PES) film to facilitate fiber taper pulling, a procedure akin to fiber drawing. One end of the glass rod was stretched to produce a fiber taper featuring a core diameter of 2 mm at the input end and 480 μm at the output end.

### The temperature distribution of the fiber endcap

The distribution of the temperature was recorded by an Infrared thermal imager (LT7-P, manufactured by Largan Precision Co., LTD.) with a measurement accuracy of ±2 °C and a resolution of 384 × 288 pixels.

### Damage measurement and calculation

The As_2_S_3_ glass (2 mm thickness, 10 mm diameter) and another one with input facet bonded to CaF_2_ lens (1 mm thickness, 10 mm diameter) were illuminated with focused laser of 2.94 μm and 4.7 μm, respectively. The diameter of the focused laser was both 500 μm. Damages occurred at the input facet of the non-bonded glass and output facet of the CaF_2_-bonded glass, under input power of 7.4 W and 11.8 W, respectively, at a wavelength of 2.94 μm. The pulsed energy densities of the fiber tapers, with and without adhesive, were calculated based on the data in Fig. 4e, f.

## Supplementary information

Supplementary Information for Breaking the Mid-Infrared Interconnection Barrier: A Robust Bonding for High-Power Optics Based on liquid-like Chalcogenide Glass

## Data Availability

Data underlying the results presented in this paper are not publicly available at this time but may be obtained from the authors upon reasonable request.

## References

[1] Armand, R. et al. Mid-infrared integrated silicon-germanium ring resonator with high Q-factor. *APL Photonics* **8**, 071301 (2023).

[2] Fang, B. et al. Bidirectional mid-infrared communications between two identical macroscopic graphene fibres. *Nat. Commun.* **11**, 6368 (2020). 33311483 10.1038/s41467-020-20033-2 PMC7733474

[3] Sato, S. I. et al. High power, high intensity CO infrared laser transmission through As_2_S_3_ glass fibers. *Appl. Phys. Lett.* **48**, 960–962 (1986).

[4] Troles, J. et al. Optical limiting behavior of infrared chalcogenide glasses. *J. Optoelectron. Adv. Mater.* **4**, 729–735 (2002).

[5] Spinelli, P., Verschuuren, M. A. & Polman, A. Broadband omnidirectional antireflection coating based on subwavelength surface Mie resonators. *Nat. Commun.* **3**, 692 (2012). 22353722 10.1038/ncomms1691 PMC3338005

[6] Yang, J. et al. Design and fabrication of broadband ultralow reflectivity black Si surfaces by laser micro/nanoprocessing. *Light Sci. Appl.* **3**, e185 (2014).

[7] Sincore, A. et al. High power single-mode delivery of mid-infrared sources through chalcogenide fiber. *Opt. Express* **26**, 7313–7323 (2018). 29609288 10.1364/OE.26.007313

[8] Siddique, R. H., Gomard, G. & Hölscher, H. The role of random nanostructures for the omnidirectional anti-reflection properties of the glasswing butterfly. *Nat. Commun.* **6**, 6909 (2015). 25901418 10.1038/ncomms7909

[9] Stroganov, V. F. & Serova, V. N. Optical adhesives: analysis, achievements, and trends in development. *Polym. Sci., Ser. D.* **12**, 410–416 (2019).

[10] Westerman, C. R., McGill, B. C. & Wilker, J. J. Sustainably sourced components to generate high-strength adhesives. *Nature* **621**, 306–311 (2023). 37704765 10.1038/s41586-023-06335-7

[11] Jinkins, K. R. et al. Thermally switchable, crystallizable oil and silicone composite adhesives for skin-interfaced wearable devices. *Sci. Adv.* **8**, eabo0537 (2022). 35687686 10.1126/sciadv.abo0537 PMC9187235

[12] Pal, S. et al. Recyclable surgical, consumer, and industrial adhesives of poly (α-lipoic acid). *Science* **385**, 877–883 (2024). 39172835 10.1126/science.ado6292

[13] Ren, H. et al. Injectable, self-healing hydrogel adhesives with firm tissue adhesion and on-demand biodegradation for sutureless wound closure. *Sci. Adv.* **9**, eadh4327 (2023). 37585520 10.1126/sciadv.adh4327 PMC10431709

[14] Petrie, E. M. *Handbook of Adhesives and Sealants*, 2nd edn. (McGraw-Hill, 2007).

[15] Savage, N. Optical adhesives. *Nat. Photonics* **3**, 418–419 (2009).

[16] Pahlevaninezhad, H. et al. Nano-optic endoscope for high-resolution optical coherence tomography in vivo. *Nat. Photonics* **12**, 540–547 (2018). 30713581 10.1038/s41566-018-0224-2 PMC6350822

[17] Broder, J. D. & Forestieri, A. F. *Improvements in Silicon Solar Cell Cover Glass Assembly and Packaging Using FEP Teflon* (National Aeronautics and Space Administration, 1970).

[18] Greenberg, S. A., McCargo, M. & Palmer, W. L. *Investigation of FEP Teflon as a Cover for Silicon Solar Cells* (Lockheed Missiles and Space Co., 1971).

[19] Griebel, J. J. et al. New infrared transmitting material via inverse vulcanization of elemental sulfur to prepare high refractive index polymers. *Adv. Mater.* **26**, 3014–3018 (2014). 24659231 10.1002/adma.201305607

[20] Nishant, A. et al. High refractive index chalcogenide hybrid inorganic/organic polymers for integrated photonics. *Adv. Optical Mater.* **10**, 2200176 (2022).

[21] Gvishi, R., Pokrass, M. & Strum, G. Optical bonding with fast sol-gel. *J. Eur. Optical Soc. Rapid* **4**, 09026 (2009).

[22] Gvishi, R. Fast sol-gel technology: from fabrication to applications. *J. Sol. Gel Sci. Technol.* **50**, 241–253 (2009).

[23] Li, Y. F. et al. Optical bonding utilizing aqueous Al_2_O_3_-P_2_O_5_-lactate sol-gel adhesives. *Ceram. Int.* **50**, 33537–33545 (2024).

[24] Gvishi, R. et al. Optical waveguide fabrication using a fast sol-gel method. *Optical Mater.* **30**, 1755–1758 (2008).

[25] Kotov, L. et al. More than 100W, 18cm Yb-doped phosphate fiber amplifier. In *Proceedings of SPIE 10897, Fiber Lasers XVI: Technology and Systems*, 108970X (SPIE, 2019).

[26] Cozic, S. et al. Splicing fluoride glass and silica optical fibers. *EPJ Web. Conf.* **215**, 04003 (2019).

[27] Xiao, L. M. et al. Fusion splicing photonic crystal fibers and conventional single-mode fibers: microhole collapse effect. *J. Lightwave Technol.* **25**, 3563–3574 (2007).

[28] Petersen, C. R. et al. Mid-infrared supercontinuum covering the 1.4-13.3 μm molecular fingerprint region using ultra-high NA chalcogenide step-index fibre. *Nat. Photonics* **8**, 830–834 (2014).

[29] Xie, Y. et al. Mid-infrared chalcogenide microfiber knot resonators. *Photonics Res.* **8**, 616–621 (2020).

[30] Flaschen, S. S., Pearson, A. D. & Northover, W. R. Low melting sulfide-halogen inorganic glasses. *J. Appl. Phys.* **31**, 219–220 (1960).

[31] Dronova, M. & Semencha, A. Water absorption and moisture resistance of As-S-Se-I glasses. *Mater. Today. Proc.* **30**, 456–461 (2020).

[32] Molla, A. R. et al. Synthesis and characterization of low T_*g*_ As-S-I chalcohalide glass for processing of raw diamonds. *Int. J. Appl. Glass Sci.* **8**, 132–135 (2017).

[33] Markov, V. A. et al. Adhesive As-S-Se-I immersion lenses for enhancing radiation characteristics of mid-IR LEDs operating in wide temperature range. *Infrared Phys. Technol.* **78**, 167–172 (2016).

[34] Dronova, M. G. & Osipov, A. Influence of Se on properties of As-S-Se-I glass for immersion mid-IR leds lenses. *Key Eng. Mater.* **822**, 811–817 (2019).

[35] Clark, R. J. H. & Rippon, D. M. The vapor phase Raman spectra, Raman band contour analyses, Coriolis constants, force constants, and values for thermodynamic functions of the trihalides of group V. *J. Mol. Spectrosc.* **52**, 58–71 (1974).

[36] Kobliska, R. J. & Solin, S. A. Raman spectrum of vitreous As_2_S_3_. *J. Non Crystalline Solids* **8**, 191–195 (1972).

[37] Nims, C. et al. Low frequency Raman spectroscopy for micron-scale and in vivo characterization of elemental sulfur in microbial samples. *Sci. Rep.* **9**, 7971 (2019). 31138888 10.1038/s41598-019-44353-6 PMC6538736

[38] Wuttig, M. et al. Revisiting the nature of chemical bonding in chalcogenides to explain and design their properties. *Adv. Mater.* **35**, 2208485 (2023). 10.1002/adma.202208485 36456187

[39] Snir, M. et al. Mechanical and optical properties of UV-curable modified acrylic adhesives. *J. Adhes.* **27**, 175–185 (1989).

[40] Adam, J. L. & Zhang, X. H. *Chalcogenide Glasses: Preparation, Properties and Applications* (Woodhead Publishing, 2014).

[41] Oettinger, P. E. Liquid coatings to decrease laser-induced surface damage in proustite. *Opt. Commun.* **13**, 431–434 (1975).

[42] Zhong, M. H. et al. Low-loss chalcogenide fiber prepared by double peeled-off extrusion. *J. Lightwave Technol.* **38**, 4533–4539 (2020).

