# Supplementary material for: Breaking the mid-infrared interconnection barrier: a robust bonding for high-power optics based on liquid-like chalcogenide glass
Source: Light Sci Appl. 2026 Mar 2;15:139. doi: 10.1038/s41377-025-02098-0 (PMC12953766; doi:10.1038/s41377-025-02098-0)
Supplement: Supplementary file 1 — Supplementary Information for Breaking the Mid-Infrared Interconnection Barrier: A Robust Bonding for High-Power Optics Based on liquid-like Chalcogenide Glass [file 41377_2025_2098_MOESM1_ESM.docx]

Supplementary Information for

Breaking the Mid-Infrared Interconnection Barrier: A Robust Bonding for High-Power Optics Based on liquid-like Chalcogenide Glass

Xian-Ge Wang,^1,2,†^ Feng Xiao,^1,†^ Yiming Du,^3^ Kai Jiao,^1^ Keke Chen,^1^ Wei Tang,^1^ Yuyang Wang,^1^ Xiang Shen,^1^ Shixun Dai,^1^ Maozhi Li,^3^ Xunsi Wang,^1,*^ Shengchuang Bai,^1^ Rongping Wang,^1,*^ Ganapathy Senthil Murugan,^4^ and Barry Luther-Davies^5^

^1^Laboratory of Infrared Material and Devices, The Research Institute of Advanced Technologies, College of Information Science and Engineering, Ningbo University, Ningbo 315211, China

^2^ College of Information and Intelligence Engineering, Zhejiang Wanli University, Ningbo 315000, China

^3^Department of Physics, Beijing Key Laboratory of Opto-Electronic Functional Materials & Micro-Nano Devices, Key Laboratory of Quantum State Construction and Manipulation (Ministry of Education), Renmin University of China, Beijing 100872, China

^4^Optoelectronics Research Centre, University of Southampton, Southampton SO17 1BJ, United Kingdom

^5^Deparment of Quantum Science and Technology, The Australia National University, Canberra 0200, Australia

^†^These authors contributed equally.

*Correspondence: wangxunsi@nbu.edu.cn; wangrongping@nbu.edu.cn

1. Supplementary Method

**1.1 Raman spectra of glasses**

We measured Raman spectra of the glasses using Renishaw PU with a 785 nm light as excitation source. The results were shown in Fig. S2.

**1.2 The ab initio Molecular Dynamics simulation of glass structure**

The ab initio molecular dynamics simulations were carried out to generate the atomic configurations of the glassy state using the projector augmented plane waves method^[1]^ within the density-functional theory as implemented in the Vienna ab initio simulation package (VASP)^[2]^. The canonical (NVT) ensemble was employed in the simulations. The simulation cell consists of 200 atoms in a cubic box with periodic boundary conditions in three directions. After well equilibrated in the liquid state at 2000 K, the system was then quenched down to 300 K with a cooling rate of 1x10^14^ K s^-1^, followed by isothermally annealing for 5000-time steps at 300 K. The last 3000 configurations were used to calculate the structural parameters. The density of the glassy state at 300 K was adopted as the measured value in experiments. The time step was set to be 3 fs, and the temperature was controlled using the Nose-Hoover thermostat^[3]^. The above simulations were performed at the Γ point only. Fig. S3 shows a snapshot of the generated atomic structure of As_20_S_60_I_20_ glass.

**1.3 The measurement of weight loss at high temperature**

The glass was initially placed in a crucible and weighed, after which it was subjected to heating in a furnace. The temperature was increased from 30 to 120 °C and maintained at 120 ℃ for 5 minutes. Following this, the glass was allowed to cool to room temperature, and the weight of the remaining glass in relation to its original weight was recorded as a measurement cycle. This procedure was repeated for a total of 37 cycles.

**1.4 Transmission spectroscopy test of liquid-like glasses**

The liquid-like glass was poured into the hollow ring mold. Then, a glass slide was used to flatten both surfaces of the sample. The transmission spectrum of the 2 mm thickness of the sample was measured by Fourier transform infrared spectrometer (America, FTIR, Nicolet 380).

**1.5 Refractive index of glasses**

The refractive indices of the liquid-like glasses were determined using an ellipsometer (IR-VASE Mark II by J.A. Woollam Inc., USA).

**1.6 Viscosity of the glass**

The viscosity of the glass adhesive with a mass of around 50 g was assessed at different temperatures using a single-cylinder rotational viscometer (RVDV-I Prime, manufactured by Brookfield Company, measuring error of 5 %). The measurement was conducted in a sealed, air-filled environment. Then, the experimental data were fitted using the Vogel-Fulcher-Tammann (VFT) equation as shown in equation of (S1)^[4]^.

$\log\eta=A+\frac{B}{(T-T_{0})}$ (S1)

where *A* and *B* are fitting coefficients, respectively, *η* is viscosity coefficient, *T* represents the temperature, and *T*_0_ denotes the Vogel temperature. For the As_20_S_60_I_20_ glass, the parameters *A*, *B*, and *T*_0_ are -1.123, 100.399, and 21.723, respectively. Similarly, for the As_15_S_45_Se_20_I_20_ glass, *A*, *B*, and *T*_0_ are -1.197, 114.124, and 18.06, respectively. The experimental data and the theoretically fitted data are interpolated as depicted in Fig. 1e in the main text.

**1.7 The theoretical calculation of Fresnel reflection on the bonded lens**

Optical loss is caused by Fresnel reflection. Formulas for deformation equation of (S2) is used to calculate the theoretical Fresnel reflectance.

$r=\left(\frac{n_{2}-n_{1}}{n_{2}+n_{1}}\right)^{2}$ (S2)

In the simulation, the wavelength is 4.7 μm, the refractive indices of CaF_2_, liquid-like glass and As_2_S_3_ are 1.4, 2.1 and 2.4, respectively. The Fresnel reflectance of surfaces F_1_-F_6_ are considered in the simulation. The final reflectivity *R* of the As_2_S_3_ glass is calculated as follows:

$R=1-(1-F_{1})\times(1-F_{2})\times(1-F_{3})\times(1-F_{4})\times(1-F_{5})\times(1-F_{6})$ (S3)

**1.8 Tensile strength measurement**

The group bonded with As_10_S_45_Se_20_I_20_ glass is connected to a specific mold for shear strength and tensile strength testing via an CMT5105 (MTS Systems Co., USA) instrument. The ASTM D3039 standard was adopted with a stretching speed of 2 mm/min.

**1.9 Bonding fibers directly with adhesive**

The coupling setup between a single-mode silica fiber with FC connector and a core diameter of approximately 10 μm and a homemade As_2_S_3_ fiber with FC connector and a length of one meter and a core diameter of approximately 80 μm prepared using a standard process. The first step is heating the adhesive and letting it melt on the surface of the FC connector on the silica fiber, and then the As_2_S_3_ fiber FC was pressed onto the surface of the liquid-like glass. After they were well aligned, the setup was cooled down in air, and was then ready for laser delivery testing.

**1.10 Design of the fiber taper**

Four kinds of tapered As-S fiber were designed to obtain the optimal mid-infrared laser output, as shown in the inset of Fig. S8a. The total diameter of the input end was 4 mm with the fiber core diameter of 2 mm, while that of the output end was 1 mm with the core diameter of 0.5 mm. Given the consistent energy preservation following uniform elongation of the fiber taper's tail end which usually results in negligible losses, our investigation focused solely on the fiber taper's transmission efficiency under conditions where the total structural length was 4 cm and the taper length was 3 cm. As indicated by the dashed curve line in Fig. S8a, within the taper region, the relationship between the end cladding diameter *D*_clad_ and the length *z* adhered to the following equation of (S4).

$D_{clad}=\left\{\begin{matrix} 4\begin{matrix} & & & \begin{matrix} \begin{matrix} & \end{matrix} & \end{matrix}\alpha=0 \end{matrix} \\ 4-3\times(\frac{z}{30})^{\alpha}\begin{matrix} & & \alpha\neq0 \end{matrix} \end{matrix}\right\}$ (S4)

here, *α* represents the taper parameter, and its value determines the extent of tapering, while *z* (mm) denotes the length of the optical fiber taper. Employing commercial software (Rsoft), we simulated the transmission efficiency *T*_1_ of the taper for four distinct curvatures: *α*_0_=0, *α*_2_=2, *α*_4_=4, *α*_8_=8. In this context, *α*_0_ signifies a straight structure without any tapering.

**References**

1. G. Kresse. et al. From ultrasoft pseudopotentials to the projector augmented-wave method. *Physical Review B* **59**, 1758 (1999).

2. G. Kresse. et al. Efficiency of ab-initio total energy calculations for metals and semiconductors using a plane-wave basis set. *Computational Materials Science* **6**, 15-50 (1996).

3. M. P. Allen. et al. Computer Simulation of Liquids (Clarendon Press, Oxford, 1987).

4. P. Koštál. et al. Viscosity of chalcogenide glass-formers. *International Materials Reviews* **65**, 63-101 (2020).

5 T. Amotchkina. et al. Characterization of e-beam evaporated Ge, YbF_3_, ZnS, and LaF_3_ thin films for laser-oriented coatings. *Applied Optics* **59**, A40-A47 (2020).

6 M. Daimon. et al. High-accuracy measurements of the refractive index and its temperature coefficient of calcium fluoride in a wide wavelength range from 138 to 2326 nm. *Applied Optics* **41**, 5275-5281 (2002).

1. Supplementary Figures


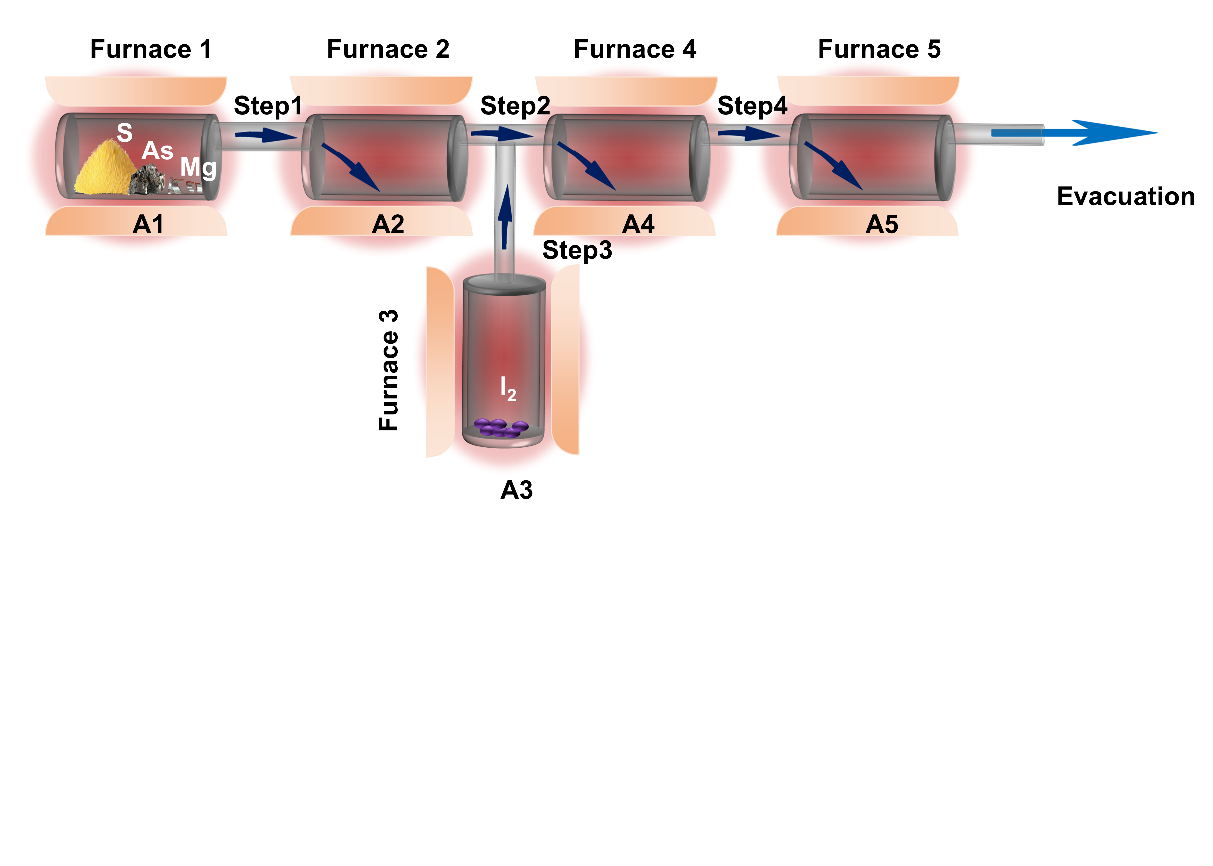


**Figure S1. Schematic diagram of glass purification.**

**
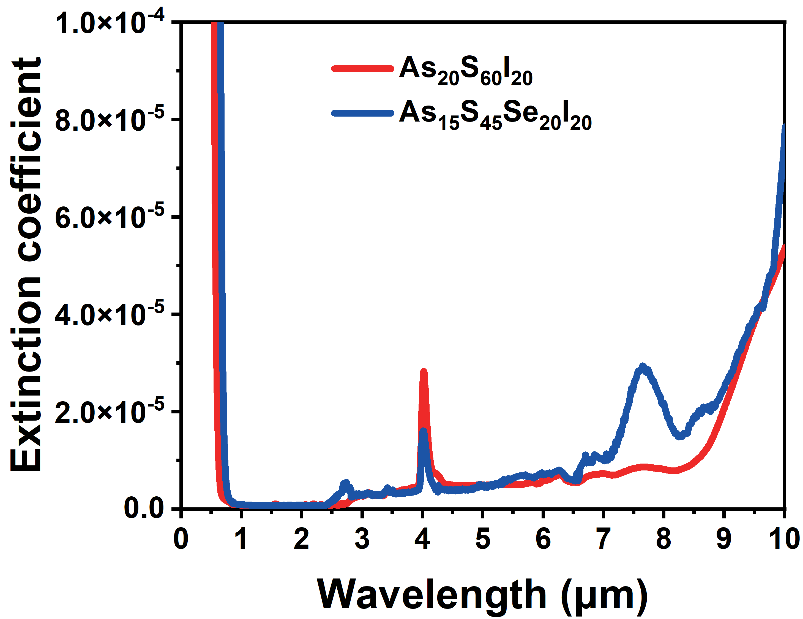
**

**Figure S2. Extinction coefficient as a function of wavelength.**

**
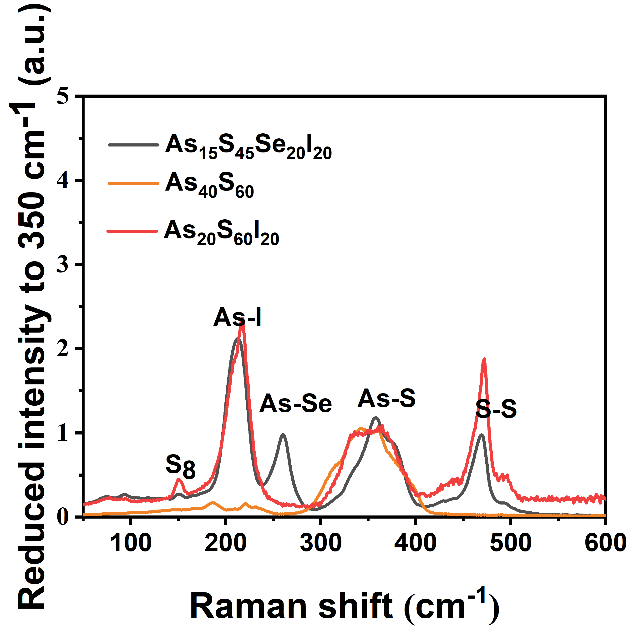
**

**Figure S3. Raman spectra of the glasses.** Reduced Raman spectra of As_20_S_60_I_20_, As_15_S_45_Se_20_I_20_ and As_40_S_60_ glasses where their intensities were normalized to that of the As-S peak at 343 cm^-1^.

**
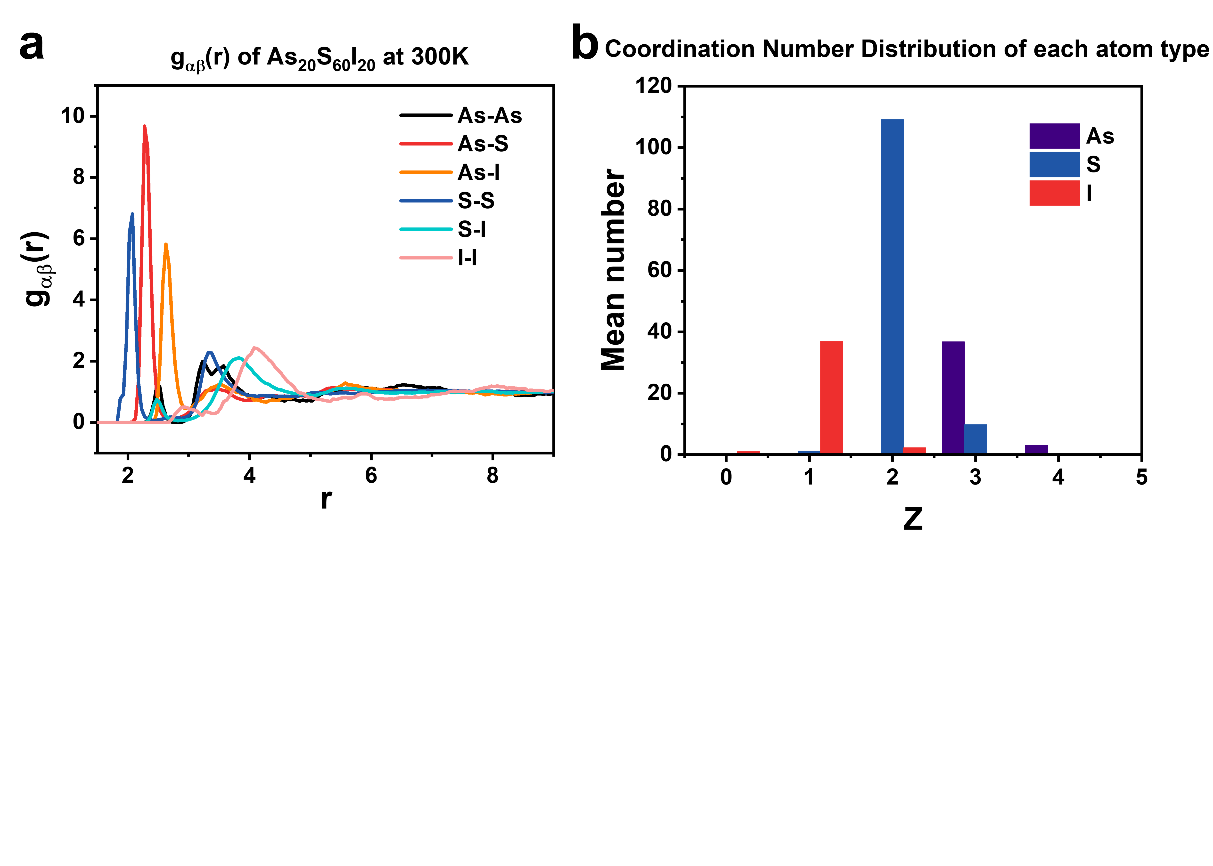
**

**Figure S4. Structural simulation of As_20_S_60_I_20_.** (a) Partial pair correlation functions of each atomic pair in As_20_S_60_I_20_ glass at 300 K averaged over 3000 atomic configurations, the insert is the simulation snapshot of the generated atomic structure of the glass. (b) Coordination number distribution of As, S, and I in As_20_S_60_I_20_ glassy samples, respectively.

**
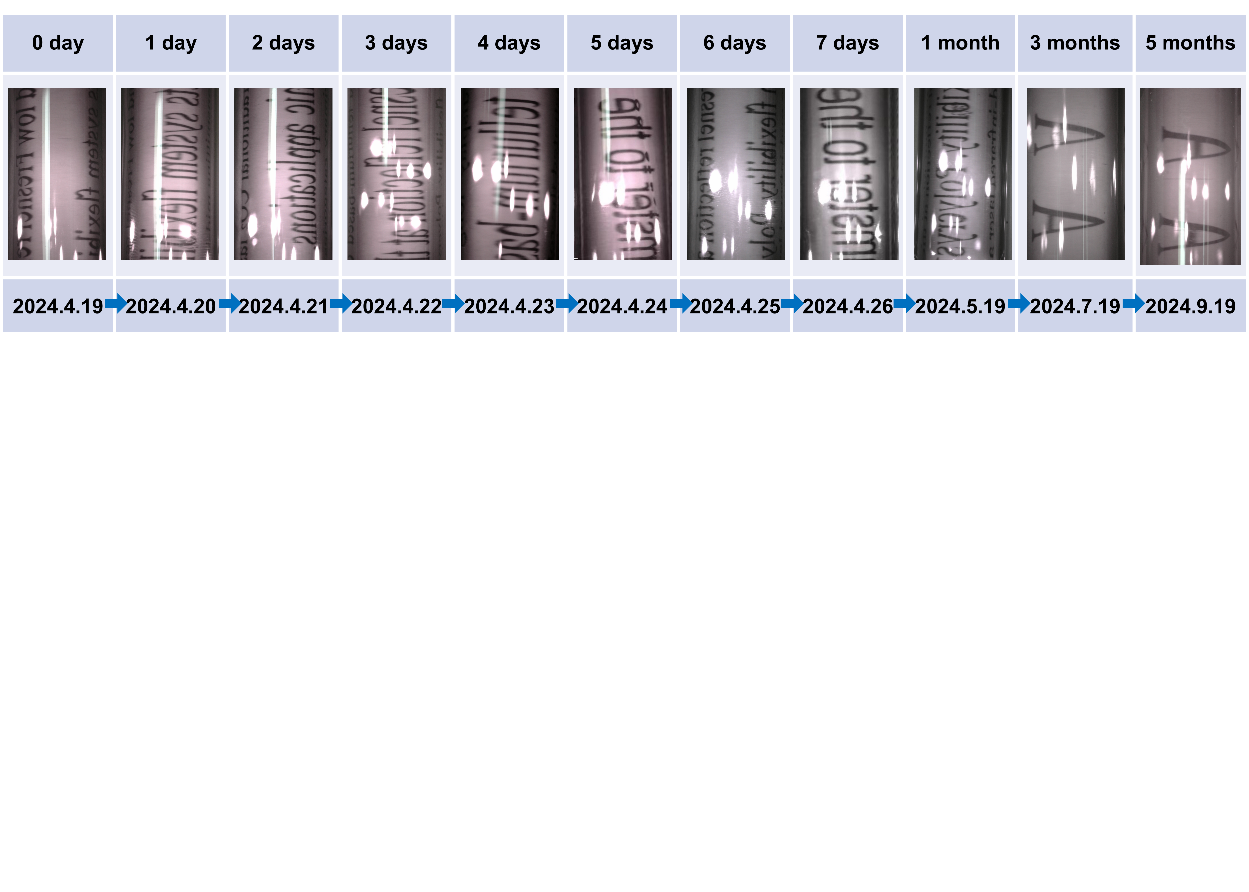
**

**Figure S5. The images of As_15_S_45_Se_20_I_20_ with different storage time recorded by near-infrared camera.**

**
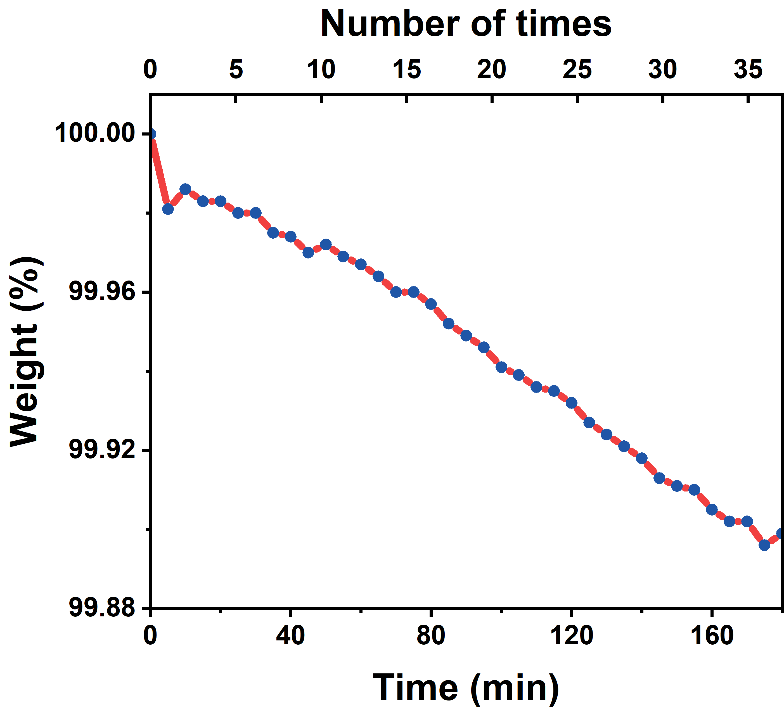
**

**Figure S6. The weight loss percent after high temperature of 120 °C.**

**
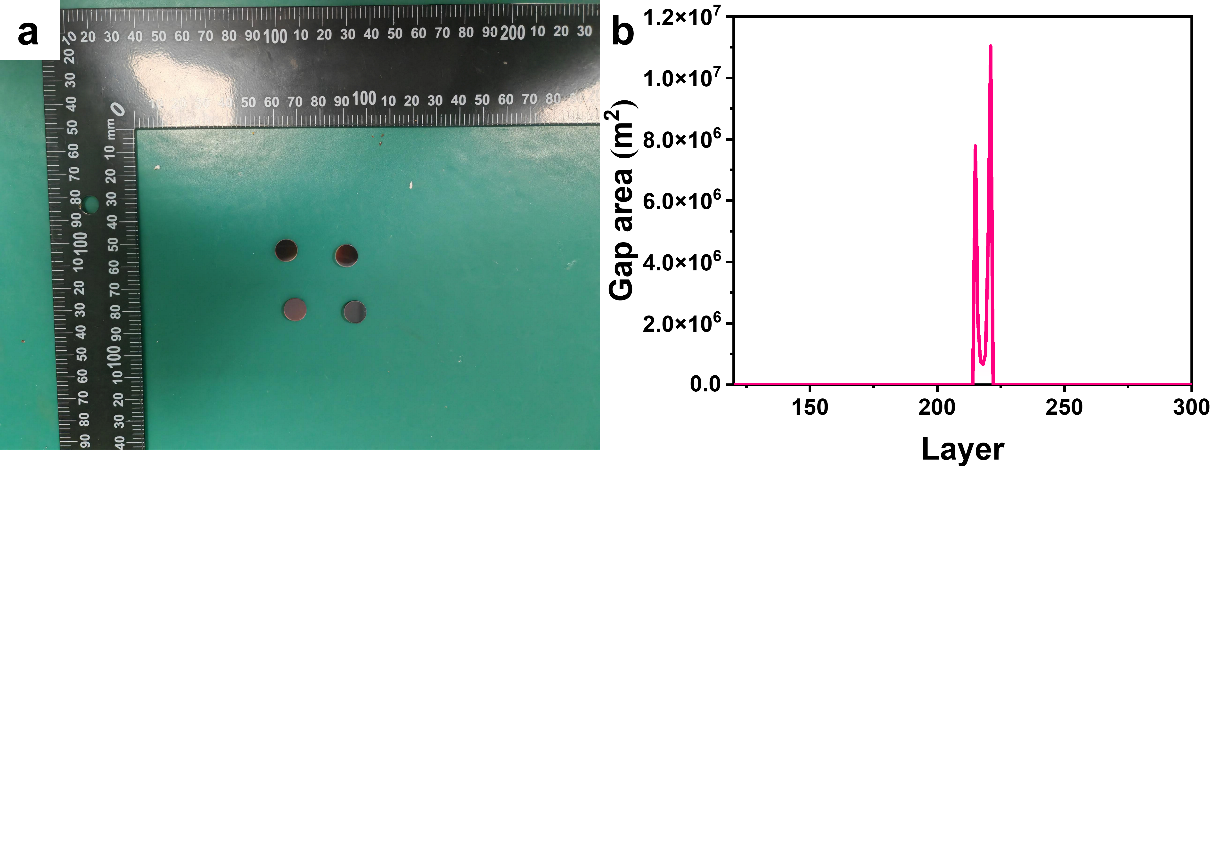
**

**Figure S7. The exposure test to the bonded CaF_2_ components.** (a) The appearances of four bonded CaF_2_ components after exposure test in accordance with the MIL-A-3920C standard. (b) The gap area of bubble at different layers.

**
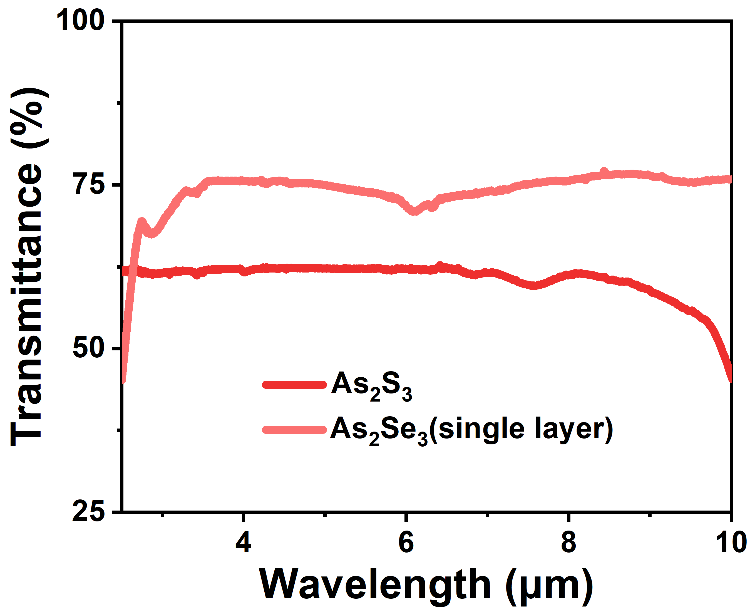
**

**Figure S8. Transmission spectra of As_2_S_3_ glass and single-sided As_2_Se_3_ coated lens.**

**
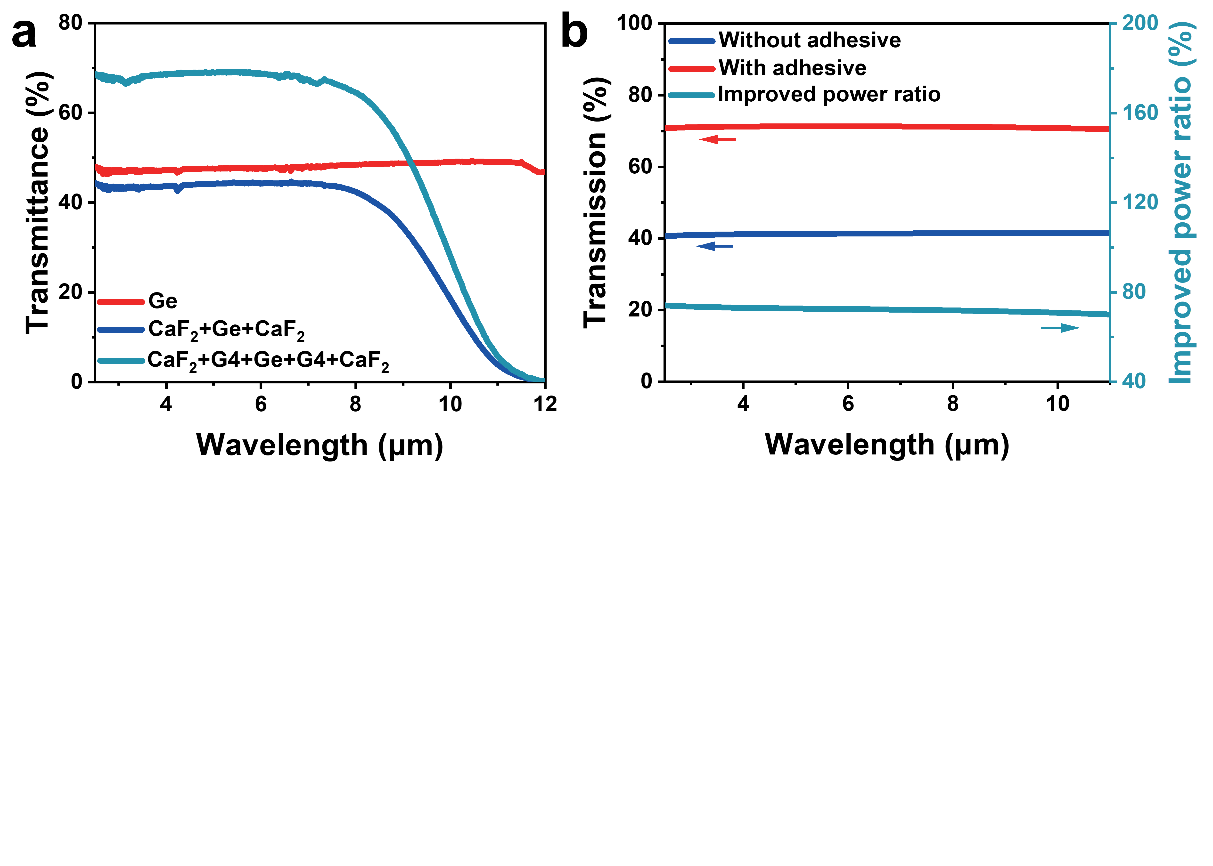
**

**Figure S9.** **Theoretical and experimental transmission of Ge lenses with and without adhesive.** (a) Theoretical Ge lens transmittance considering only Fresnel reflections. (b) Experimental FTIR transmittance of lenses with and without the optical adhesive. (The refractive index data are from References [5] and [6].)

**
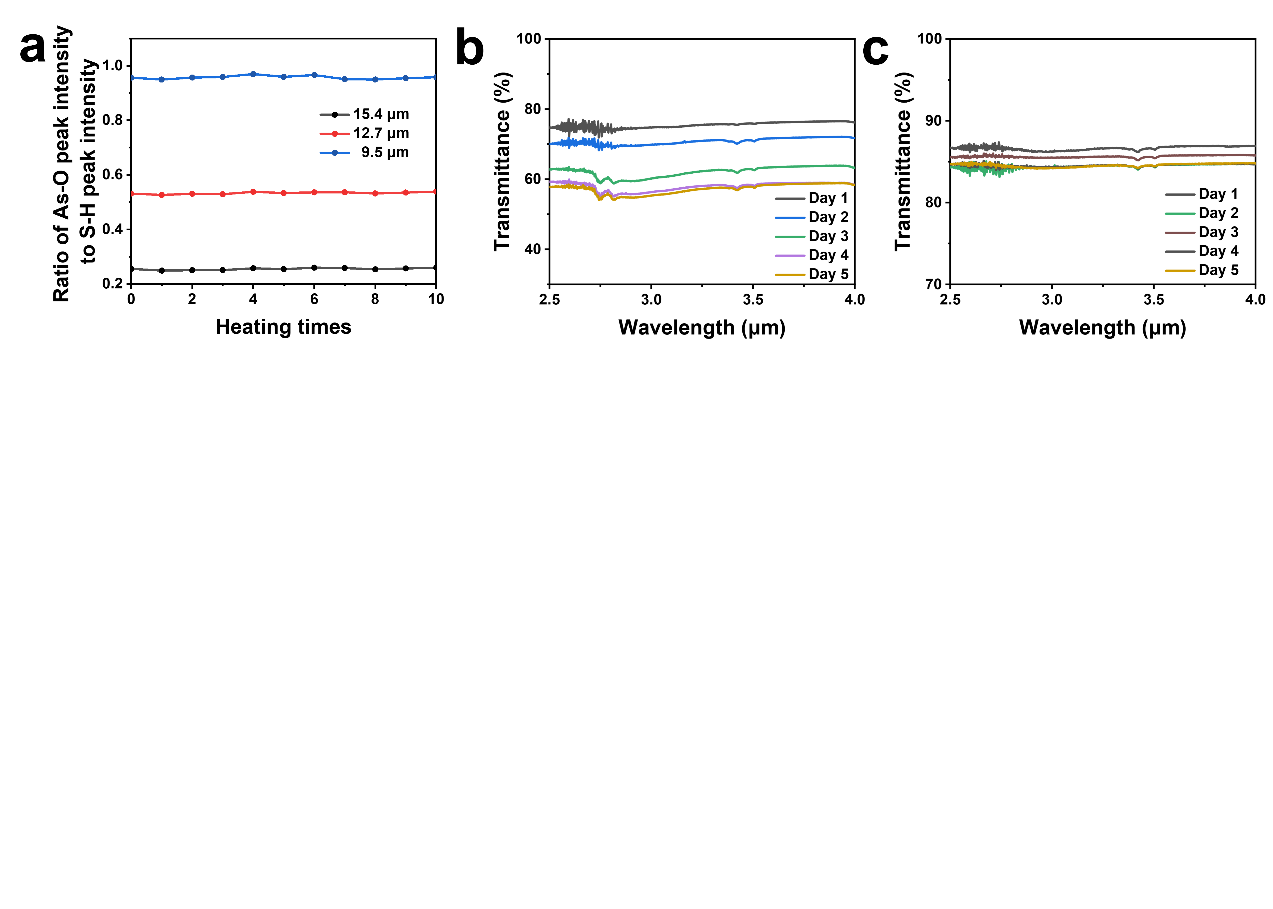
**

**Figure S10** **Oxidation and moisture resistance tests of As–S(Se)–I glass**. (a) Variation in As–O peak relative intensity after repeated heating cycles. (b) Transmittance of As–S(Se)–I glass with one surface exposed to water for 5 days. (c) Transmittance of fully encapsulated bonded components after 5 days of water immersion.

**
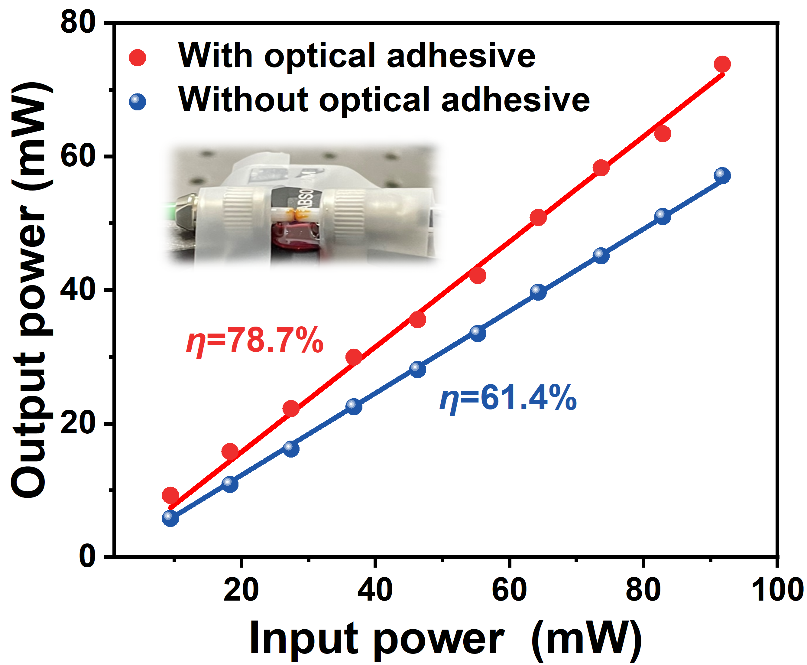
**

**Figure S11. Laser delivery of the fiber at 1.55 µm, with and without optical adhesive.** Without optical adhesive, the transmission (slope) of the FC with free-space coupling is only 61.4%. The inset is a physical picture of using glass adhesive to connect two FC connectors, and in this case, the transmission efficiency increases to 78.7%.

**
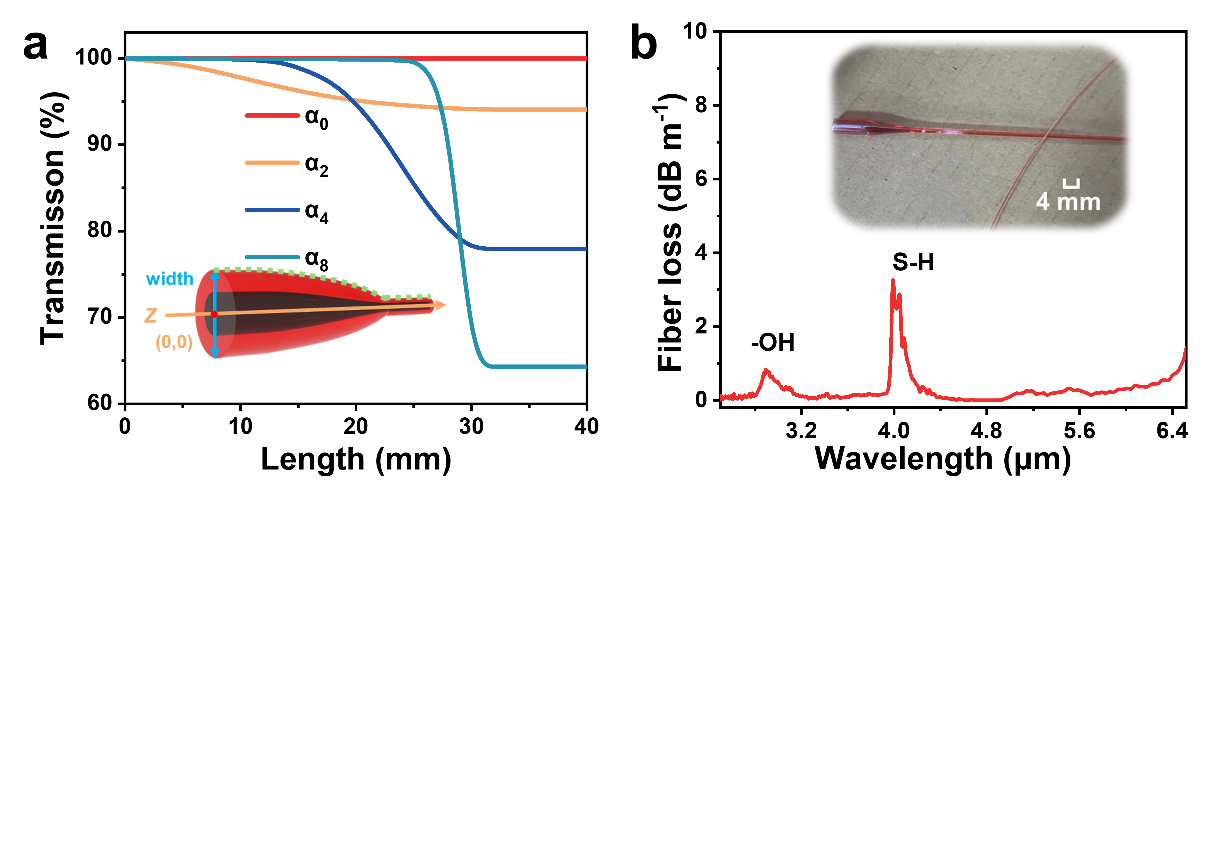
**

**Figure S12. Fiber taper design and actual characteristics.** (a) Transmission of the 4 types of tapers (design of fiber taper structure insert). Clearly, structural losses escalate with increasing degrees of taper. The maximum transmission rate achieved for the fiber taper stands at 94% as shown in the yellow curve. (b) Optical loss of the fiber taper (the As-S fiber taper insert). Notably, there are discernible absorption peaks occurring around 2.9 μm and 4.1 μm, with losses amounting to 0.83 dB/m and 3.27 dB m^-1^, respectively. At 4.7 μm, the loss approaches approximately 0.1 dB m^-1^.

**
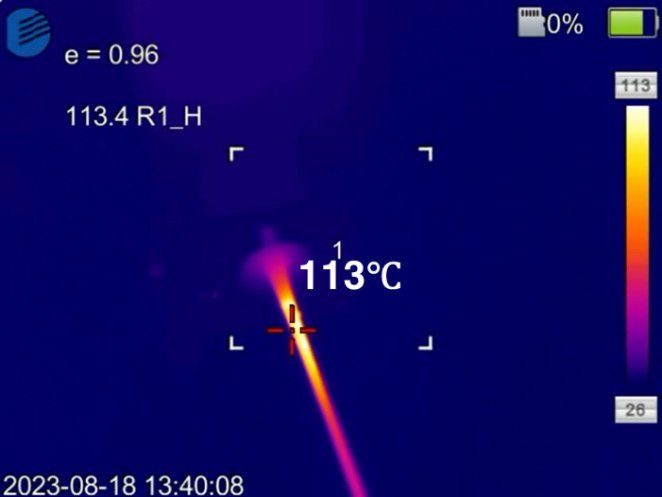
**

**Figure S13. The distribution of the temperature of fiber endcap by an Infrared Thermal Imager.**

**
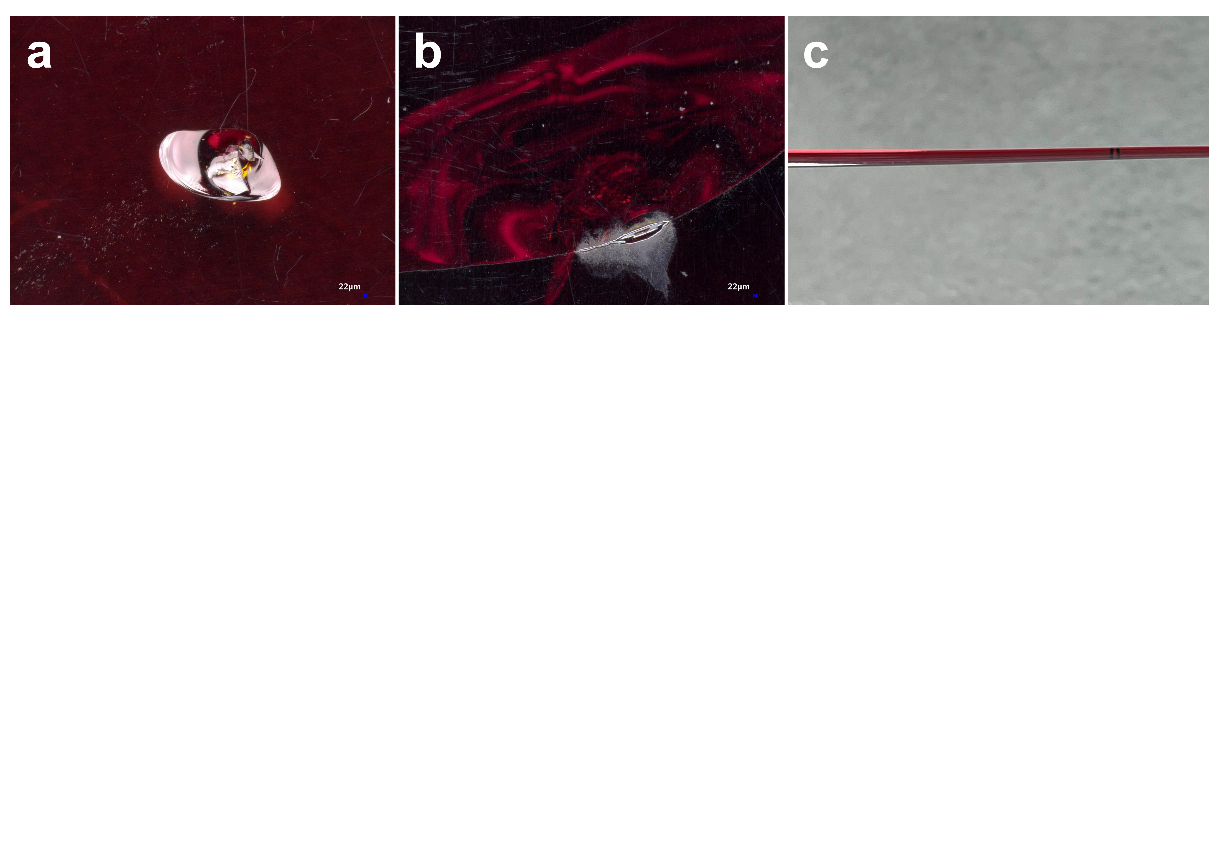
**

**Figure S14. Typical damages in lens and fiber taper.** (a) Damage in the input facet of As_2_S_3_ lens without adhesive. (b) Damage in the output facet of As_2_S_3_ lens with adhesive. (c) Damage in the taper of As_2_S_3_ fiber.

1. Supplementary Tables

**Table S1. The *T*_g_ and *n* of different glass composition**

| Glass samples | Composition (at. %) | *T*_g_ (°C) | *n*@4.7 μm | State under room temperature |
| --- | --- | --- | --- | --- |
| G1 | As_20_S_60_I_20_ | 10.61/116.71 | 2.10 | Liquid-like |
| G2 | As_15_S_15_Se_50_I_20_ | 28.91 | 2.34 | Liquid-like |
| G3 | As_15_S_30_Se_35_I_20_ | 19.79 | 2.21 | Liquid-like |
| G4 | As_15_S_45_Se_20_I_20_ | 8.85 | 2.10 | Liquid-like |

**Table S2. Laser damage thresholds of glasses and fiber tapers with or without adhesive**

| Types | Without optical adhesive | | | With optical adhesive | | | Absorption |
| --- | --- | --- | --- | --- | --- | --- | --- |
|  | Input^*^  (J/cm^2^) | Output^*^  (J/cm^2^) | Damage Position | Input^*^  (J/cm^2^) | Output^*^  (J/cm^2^) | Damage Position |  |
| Glass | 253.1 | 186.4 | Input  facet | 402.0 | 324.1 | Output  facet | OH^-^ impurity |
| Fiber taper | 39.3 | 85.0 | Taper  region | 49.2 | 140.1 | Taper  region | OH^-^ impurity & Coating Polymer |

Notes: ^*^is the pulsed energy density
